# The mGluR5‐mediated Arc activation protects against experimental traumatic brain injury in rats

**DOI:** 10.1111/cns.14695

**Published:** 2024-08-06

**Authors:** Tao Chen, Yun‐Fei Li, Xu Ren, Yu‐Hai Wang

**Affiliations:** ^1^ Department of Neurosurgery Wuxi Taihu Hospital, Wuxi Clinical Medical School of Anhui Medical University Wuxi China

**Keywords:** Arc, glutamate receptor, mGluR5, post‐synaptic density, traumatic brain injury

## Abstract

**Introduction:**

Traumatic brain injury (TBI) is a complex pathophysiological process, and increasing attention has been paid to the important role of post‐synaptic density (PSD) proteins, such as glutamate receptors. Our previous study showed that a PSD protein Arc/Arg3.1 (Arc) regulates endoplasmic reticulum (ER) stress and neuronal necroptosis in traumatic injury in vitro.

**Aim:**

In this study, we investigated the expression, regulation and biological function of Arc in both in vivo and in vitro experimental TBI models.

**Results:**

Traumatic neuronal injury (TNI) induced a temporal upregulation of Arc in cortical neurons, while TBI resulted in sustained increase in Arc expression up to 24 h in rats. The increased expression of Arc was mediated by the activity of metabotropic glutamate receptor 5 (mGluR5), but not dependent on the intracellular calcium (Ca^2+^) release. By using inhibitors and antagonists, we found that TNI regulates Arc expression via G_q_ protein and protein turnover. In addition, overexpression of Arc protects against TBI‐induced neuronal injury and motor dysfunction both in vivo and in vitro, whereas the long‐term cognitive function was not altered. To determine the role of Arc in mGluR5‐induced protection, lentivirus‐mediated short hairpin RNA (shRNA) transfection was performed to knockdown Arc expression. The mGluR5 agonist (RS)‐2‐chloro‐5‐hydroxyphenylglycine (CHPG)‐induced protection against TBI was partially prevented by Arc knockdown. Furthermore, the CHPG‐induced attenuation of Ca^2+^ influx after TNI was dependent on Arc activation and followed regulation of AMPAR subunits. The results of Co‐IP and Ca^2+^ imaging showed that the Arc‐Homer1 interaction contributes to the CHPG‐induced regulation of intracellular Ca^2+^ release.

**Conclusion:**

In summary, the present data indicate that the mGluR5‐mediated Arc activation is a protective mechanism that attenuates neurotoxicity following TBI through the regulation of intracellular Ca^2+^ hemostasis. The AMPAR‐associated Ca^2+^ influx and ER Ca^2+^ release induced by Homer1‐IP_3_R pathway might be involved in this protection.

## Graphical abstract

The mGluR5‐mediated Arc induction might be an endogenous neuroprotective mechanism following TBI, and the AMPAR‐associated Ca^2+^ influx and ER Ca^2+^ release induced by Homer1‐IP_3_R pathway were involved in this protection.

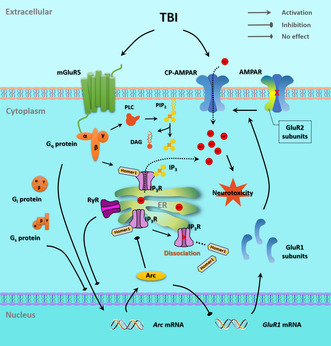

## 1 INTRODUCTION

More than 9 million people suffer from traumatic brain injury (TBI) each year in China, most of which are caused by traffic accidents and falls.^1^, ^2^ With the improvements in traffic safety, emergency equipment and specialized neurosurgical intensive care units (ICU), TBI patients are getting better prognosis.^3^ However, the molecular mechanisms underlying TBI‐induced neuronal injury and neurological deficits are not fully understood. It is currently accepted that TBI should be considered as not a single pathophysiological event, but a cascade that involves complex signaling pathways.^4^, ^5^ Thus, finding new strategies to target multiple detrimental cascades after TBI are urgently needed.

Increase in extracellular glutamate, the major excitatory neurotransmitter in the mammalian central nervous system (CNS), is a key event following TBI, and the loss of glutamate homeostasis plays an important role in neuronal damage.^6^ In human, elevated levels of glutamate in cerebrospinal fluid (CSF) can be observed after TBI, which might persist for days and weeks.^7^, ^8^ Glutamate induces neurotoxicity through activation of ionotropic glutamate receptors (iGluRs), which mediate direct and fast information transfer, and metabotropic glutamate receptors (mGluRs), which control neuronal excitability and neurotransmitter release.^9^ Many previous studies have demonstrated that the agonists and antagonists of various glutamate receptors have therapeutic potentials for TBI. Our previous experiments also showed that the mGluR5 positive modulators attenuated traumatic neuronal injury,^10^, ^11^ while the α‐amino‐3‐hydroxy‐5‐methyl‐4‐isoxazole propionate (AMPA) receptor (AMPAR) antagonist perampanel protected against TBI‐induced neurological deficits.^12^ However, the exact mechanisms underlying these effects, as well as the complex crosstalk between different glutamate receptors are not fully determined.

Intracellular calcium (Ca^2+^) overload is a key mechanism that contributes to glutamate‐induced neurotoxicity after TBI, and it can be caused by the Ca^2+^ influx from the extracellular space via iGluRs or by the Ca^2+^ rise by intracellular Ca^2+^ release from the endoplasmic reticulum (ER) through mGluRs, especially the group I mGluRs (mGluR1 and mGluR5).^13^, ^14^ For the regulation of intracellular Ca^2+^ homeostasis, various post‐synaptic density (PSD) proteins, such as Homer, Shank and Arc/Arg3.1 (Arc), are needed to form a polymeric network structure as an assembly platform for the binding and crosstalk of the glutamate receptors.^15^, ^16^ Arc is coded by the brain‐specific immediate‐early gene (IEG) *Arc* and is selectively expressed in post‐synaptic density (PSD) in neuronal cells.^17^ Previous studies showed that the expression of Arc was highly dynamic, and dysregulation of Arc and related signaling was associated with cognitive disorders, including autism and Alzheimer's disease.^18^ Our previous data showed that glutamate induced rapid induction of Arc via the N‐methyl‐d‐aspartate (NMDA) receptor (NMDAR)‐mediated phosphorylation of extracellular signal‐regulated kinase (ERK) and cAMP‐response element binding protein (CREB).^19^ More recently, we found that knockdown of Arc aggravates traumatic neuronal injury via mGluR1‐mediated ER stress and necroptosis.^20^ However, the expression and biological function of Arc in neuronal injury after TBI has not been determined. In this study, we investigated the expression and regulation of Arc protein in both in vitro and in vivo experimental TBI models, and we also determined the role of Arc as well as the potential molecular mechanism with focus on glutamate receptors cascades.

## 2 MATERIALS AND METHODS

### 2.1 Animals and agents

Adult male Sprague–Dawley (SD) rats (weighting approximately 300 g) and pregnant female SD rats (E16‐18) were obtained from the Animal Experimental Center of Anhui Medical University. All animal procedures were approved and supervised by the Animal Ethics Committee of Anhui Medical University. The mGluR1 antagonist 1‐aminoindan‐1,5‐dicarboxylic acid (AIDA, #A254), the mGluR5 antagonist methyl‐6‐(phenylethynyl)‐pyridine (MPEP, #M5435), the mGluR1 agonist (S)‐3,5‐dihydroxyphenylglycine (DHPG, #D3689), the IP_3_R antagonist xestospongin C (Xes, #X2628), the G_i_ protein inhibitor pertussis toxin (PTX, #P7208), the G_s_ protein inhibitor Cholera toxin (CTX, #SAE0069), the G_q_ protein inhibitor 1‐[6‐[[17b‐3‐methoxyestra‐1,3,5(10)‐trien‐17‐yl]amino]exyl]‐1H‐pyrrole‐2,5‐dione (U‐73122, #U6756) and the proteasome inhibitor MG132 (#SML1135) were purchased from Sigma (St. Louis, MO, USA). The mGluR5 agonist (RS)‐2‐chloro‐5‐hydroxyphenylglycine (CHPG, #ab120039), the group II mGluRs antagonist (2S)‐2‐amino‐2‐{(1S,2S)‐2‐carboxycycloprop‐1‐yl}‐3‐(xanth‐9‐yl)propanoic acid (LY341495, #ab144500), the RyR antagonist ryanodine (#ab120083), the protein synthesis inhibitor cycloheximide (CHX, #ab120093) and the Ca^2+^‐permeable AMPAR antagonist 1‐naphthyl acetyl spermine (NASPM, #ab146810) were obtained from Abcam (Shanghai, China).

### 2.2 Primary culture of cortical neurons

Cortical neurons were cultured from pregnant female SD rats using our previously described methods.^16^ The cultured neurons were seeded on 1.5 cm glass slides pre‐coated with poly‐l‐lysine (PLL, 50 μg/mL) for immunostaining and Ca^2+^ imaging.

### 2.3 Experimental models

To mimic neuronal injury following TBI, the TNI model was performed according to our previously published method.^21^ For in vivo experiments, TBI was induced by using a controlled cortical impact (CCI) model in according with our previously detailed methods.^12^

### 2.4 Immunostaining

For in vitro experiments, the neurons seeded on coverslips were fixed with 4% paraformaldehyde in PBS, treated with 0.1% Triton X‐100, and then were blocked by 5% bovine serum albumin (BSA, Gibco, Gaithersburg, MD, USA). For in vivo experiments, 20 μm‐thick slices from the perfused brains were used for immunostaining. The antibodies and dilutions used were: Arc (sc‐17839, Santa Cruz, 1:100), MAP‐2 (#8707, Cell Signaling, 1:200), Alexa Fluor 594 (Red, 1:500) and Alexa Fluor 488 (Green, 1:500) secondary antibodies. TUNEL staining was performed by labeling with fluorescein TUNEL reagent mixture for 60 min at 37°C according to the manufacturer's suggested protocol (Roche, Basel, CH). DAPI (10 μg/mL) was used to stain the nuclei, and the pictures were obtained using a Zeiss fluorescent imaging microscope (Carl Zeiss, Thornwood, NY, USA).

### 2.5 Ca^2+^ imaging

Ca^2+^ imaging was performed using the Ca^2+^ indicator Fura‐2 AM (Molecular Probes, Eugene, OR) to measure the intracellular Ca^2+^ concentrations.^20^ The neurons cultured in coverslips were loaded with 5 μM Fura‐2 AM in HBSS solution for 30 min and equilibrated lucifugally for 30 min. Cells were excited at 345 and 385 nm using a confocal laser scanning microscope, and the emission fluorescence at 510 nm was recorded. The fluorescence values were then plotted against time and shown as *F*/*F*_0_.

### 2.6 Brain water content

Brain edema was determined by measuring brain water content with the wet‐dry method. After rats were anesthetized and sacrificed by decapitation, the brains were quickly removed and separated through the inter‐hemispheric fistula into left and right hemispheres. Tissue samples from injured hemispheres were weighed immediately to get wet weight. After drying in an oven for 48 h at 100°C, the tissues were re‐weighed to get the dry weight. Brain water content was then calculated using the following formula: % H_2_O = (1 − dry weight/wet weight) × 100%.

### 2.7 Measurement of neurological dysfunction

#### 2.7.1 Beam walk task

Twenty‐four hours before TBI and 1, 3, and 7 days after TBI, beam walk task was performed to determine motor coordination. Briefly, animals were placed on the end of a 100 cm beam (1.5 cm wide) suspended 60 cm above the ground. The rats' home cage was placed on the far end of the beam to attract the animals. Begin the timing when the rat was securely positioned on the beam, and record the time to traverse the beam for a maximum of 60 s. If the rat failed to balance on its own or cannot cross the beam within 60 s, the time was recorded as 60 s.

#### 2.7.2 Morris water maze (MWM) task

The MWM test was used to evaluate spatial learning and memory function as previously described.^12^ For each training trial, the rat was randomly placed in 1 of the 4 quadrants and allowed to swim freely for 120 s or until it found the platform. If the rat was unable to find the platform within 120 s, it was gently guided to the platform by the experimenter. The latency time to reach the hidden platform was recorded. Two hours after the training trials, a probe trial was performed to test spatial memory function. The platform was removed and the rat was allowed to search freely for 120 s. The time when the rat was in the former platform location was recorded to assess spatial memory.

### 2.8 Cell viability

Cell viability was measured by the WST‐1 method using a kit according to the manufacture's protocol (Jianchen Bioengineer Institute, Nanjing, Jiangsu, China).^22^ The results were expressed as a percentage of the control value.

### 2.9 Lactate dehydrogenase (LDH) release

The neurotoxicity in vitro was determined by measuring LDH release using a kit according to the manufacture's protocol (Jiancheng Bioengineer Institute, Nanjing, Jiangsu, China).^23^ The results were expressed as a fold of the control value.

### 2.10 Nitric oxide (NO) production

Intracellular NO concentration was estimated using 4‐amino‐5‐methylamino‐2′,7′‐difluorofluorescein (DAFFM) diacetate according to the manufacturer's instructions (Molecular Probes, Eugene, OR, USA).^23^

### 2.11 Co‐inmunoprecipitation (Co‐IP)

Co‐IP was performed by following a previously published procedure.^24^ Briefly, cells were harvested in a PBS‐based buffer containing 1 mM EGTA, 1 mM EDTA, 1 mM PMSF, 2 μg/mL aprotinin, 20 μg/mL pepstatin A and 20 μg/mL leupeptin. After centrifugation, cells were lysed and solubilized in harvesting buffer containing 0.1% SDS and 0.8% Triton X‐100. An equal amount of the lysate (one‐tenth) was reserved for input loading, and the remainder was incubated with protein‐A/G beads for 1 h at 4°C to remove any nonspecifically bound proteins. The supernatant was collected and centrifuged and then incubated with the antibody against Homer1 for 1 h at 4°C. The complex was precipitated with 50% protein A/G beads slurry. Proteins were separated and detected on immunoblots with antibodies against Arc, IP_3_R and Homer1.

### 2.12 Arc knockdown and overexpression

The lentivirus‐mediated knockdown and overexpression were used to determine the role of Arc, and all lentiviruses were developed and obtained from GeneChem Company (Shanghai, China). To develop the short hairpin RNA (shRNA) lentiviruses, an siRNA oligo (CCAACGUGAUCCUGCAGAU) was subcloned into a GV248 lentiviral vector (hU6‐MCS‐Ubiquitin‐EGFPIRES‐puromycin). To develop overexpression lentiviruses, cDNA of Arc was subcloned into a G492 lentiviral vector (Ubi‐MCS‐3FLAGCBh‐gcGFP‐IRES‐puromycin). The cultured neurons were used for transfection on DIV 12–14.

### 2.13 Western blot analysis

A standard western blot assay was performed using the following primary antibodies: Arc (sc‐17839, Santa Cruz, 1:300), GluA1 (#13185, Cell Signaling, 1:1000), GluA2 (#13607, Cell Signaling, 1:1000), mGluR5 (#ab71316, Abcam, 1:500), Homer1 (#ab184955, Abcam, 1:500), IP_3_R (#ab108517, Abcam, 1:800) and β‐actin (ab8226, Abcam, 1:2000). After incubation with secondary antibodies for 1 h, the bands were visualized by using chemiluminescent detection system.

### 2.14 Statistical analysis

Statistical analysis was performed using SPSS 16.0, a statistical software package. The Kolmogorov–Smirnov test was used to confirm the data distribution as normal/Gaussian distribution. Significant differences between experiments were assessed by univariate ANOVA (more than two groups) followed by Bonferroni's multiple comparison or unpaired t tests (two groups). A value of *p* < 0.05 was considered statistically significant.

## 3 RESULTS

### 3.1 TBI upregulates Arc expression via mGluR5 in vivo

**FIGURE 1:**
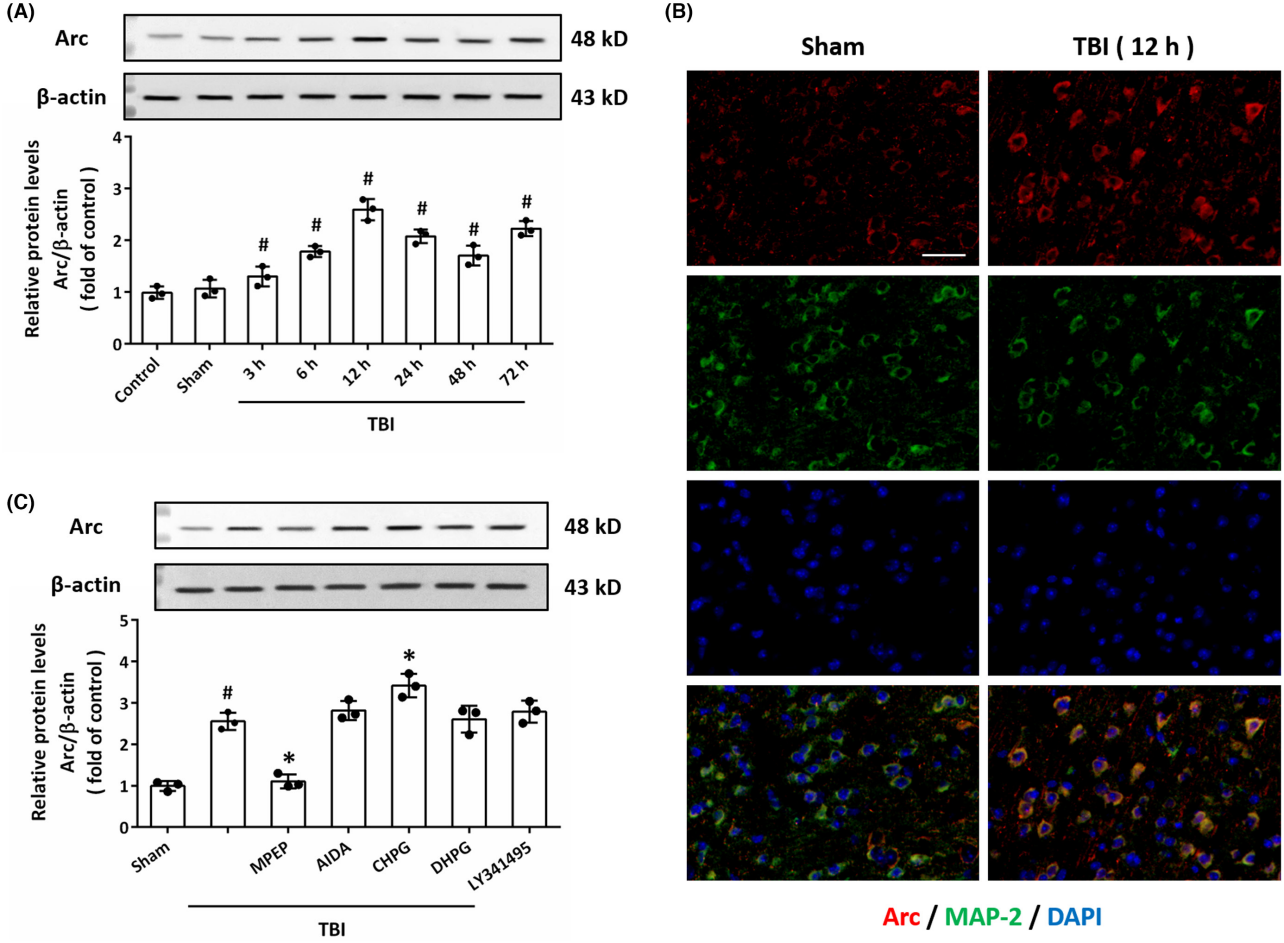
TBI upregulates Arc expression via mGluR5 in vivo. (A) Western blot and quantification show the increased levels of Arc from 3 to 72 h after TBI in the cortex in rats. (B) Immunostaining with anti‐Arc antibody (red) and anti‐MAP‐2 antibody (green) show the increased levels of Arc in neurons at 12 h after TBI. Scale bar, 50 μm. (C) Western blot and quantification show that TBI upregulated Arc through mGluR5, but not via mGluR1 in vivo. The data were represented as mean ± SEM. ^#^ *p* < 0.05 versus Sham group and *p < 0.05 versus TBI group.

To investigate the effect of TBI on Arc expression in vivo, animals were injured by TBI, and western blot was performed at different time points using Arc antibody (Figure 1A). The results showed that TBI significantly upregulated Arc levels from 3 to 72 h, the Arc expression peaked at 12 h post‐TBI. As shown in Figure 1B, the expression of Arc was mainly observed in neurons, and the subcellular distribution of Arc was not altered by TBI at 12 h. To investigate the potential role of group I mGluRs in TBI‐induced Arc expression, animals were treated with agonists and antagonists of mGluR1 or mGluR5 before TBI (Figure 1C). The results of western blot showed that TBI‐induced Arc upregulation was nullified by the mGluR5 antagonist MPEP (100 nmol) and promoted by mGluR5 agonist CHPG (100 μM). However, the mGluR1 antagonist AIDA (30 μM), the mGluR1 agonist DHPG (30 μM), or the group II mGluRs antagonist LY341495 (2 μM) had no effect on Arc expression after TBI.

### 3.2 TNI induces Arc expression via mGluR5 in vitro

**FIGURE 2:**
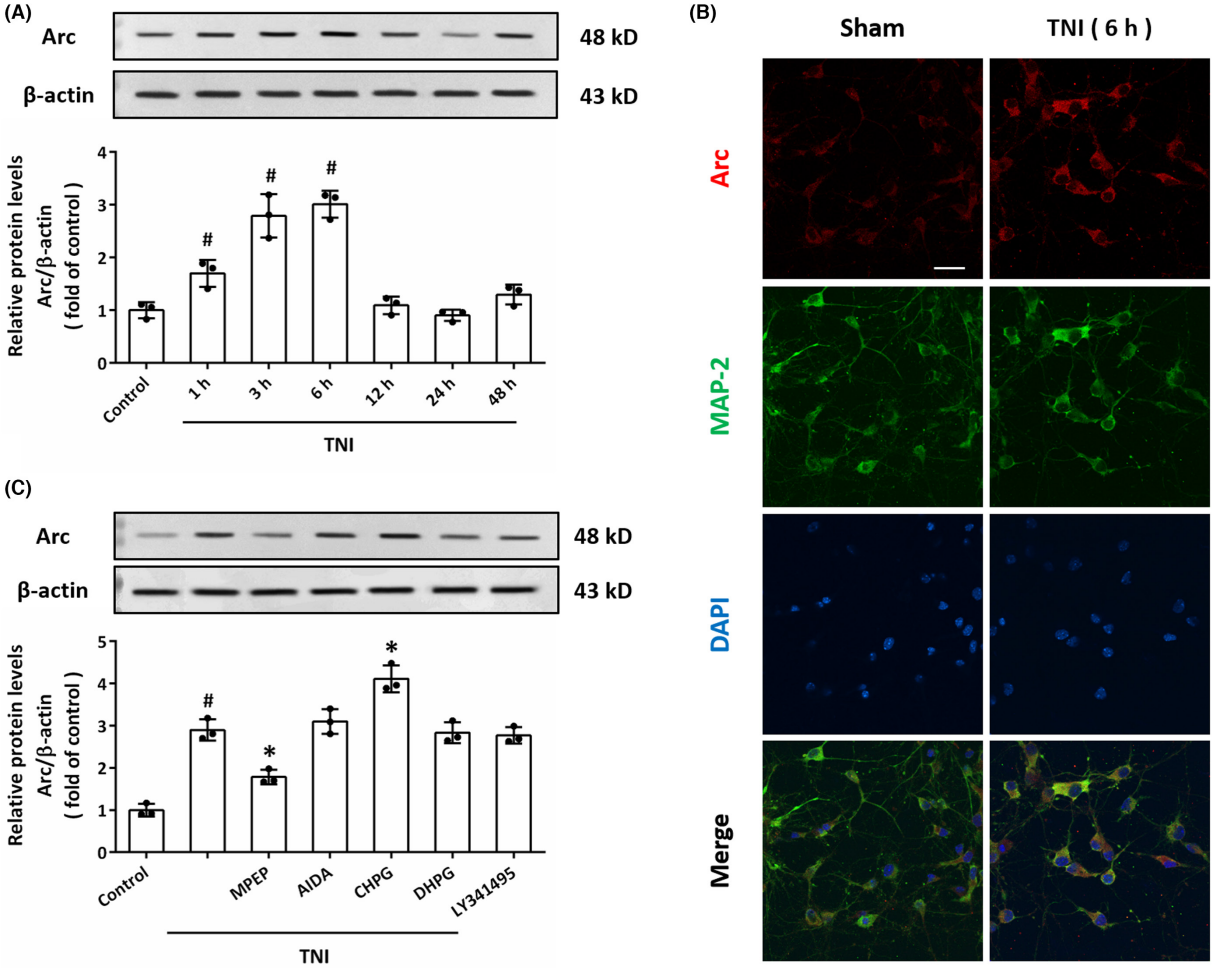
TNI induces Arc expression via mGluR5 in vitro. (A) Western blot and quantification show the increased levels of Arc from 1 to 6 h after TNI in cultured neurons. (B) Immunostaining with anti‐Arc antibody (red) and anti‐MAP‐2 antibody (green) show the increased levels of Arc in neurons at 6 h after TNI. Scale bar, 50 μm. (C) Western blot and quantification show that TNI upregulated Arc through mGluR5, but not via mGluR1 in vitro. The data were represented as mean ± SEM. ^#^ *p* < 0.05 versus Control group and *p < 0.05 versus TNI group.

To investigate the effect of TBI on Arc expression in vitro, cultured cortical neurons were used to establish a TNI model, an in vitro model of TBI. The results of western blot showed that TNI induced a temporal upregulation of Arc from 1 to 3 h (Figure 2A). As shown in Figure 2B, Arc protein was observed in cytoplasm and dendritic spines in cultured neurons, and the subcellular distribution of Arc was not altered by TNI at 6 h. Next, we also investigated the role of group I mGluRs in Arc expression in vitro by using agonists and antagonists (Figure 2C). The results of western blot showed that TNI‐induced Arc upregulation was attenuated by MPEP (1 μM) and enhanced by CHPG (1 mM). In congruent with in vivo results, AIDA (300 μM), DHPG (300 μM) or LY341495 (20 μM) had no effect on Arc expression after TNI.

### 3.3 mGluR5 regulates Arc expression through G proteins

**FIGURE 3:**
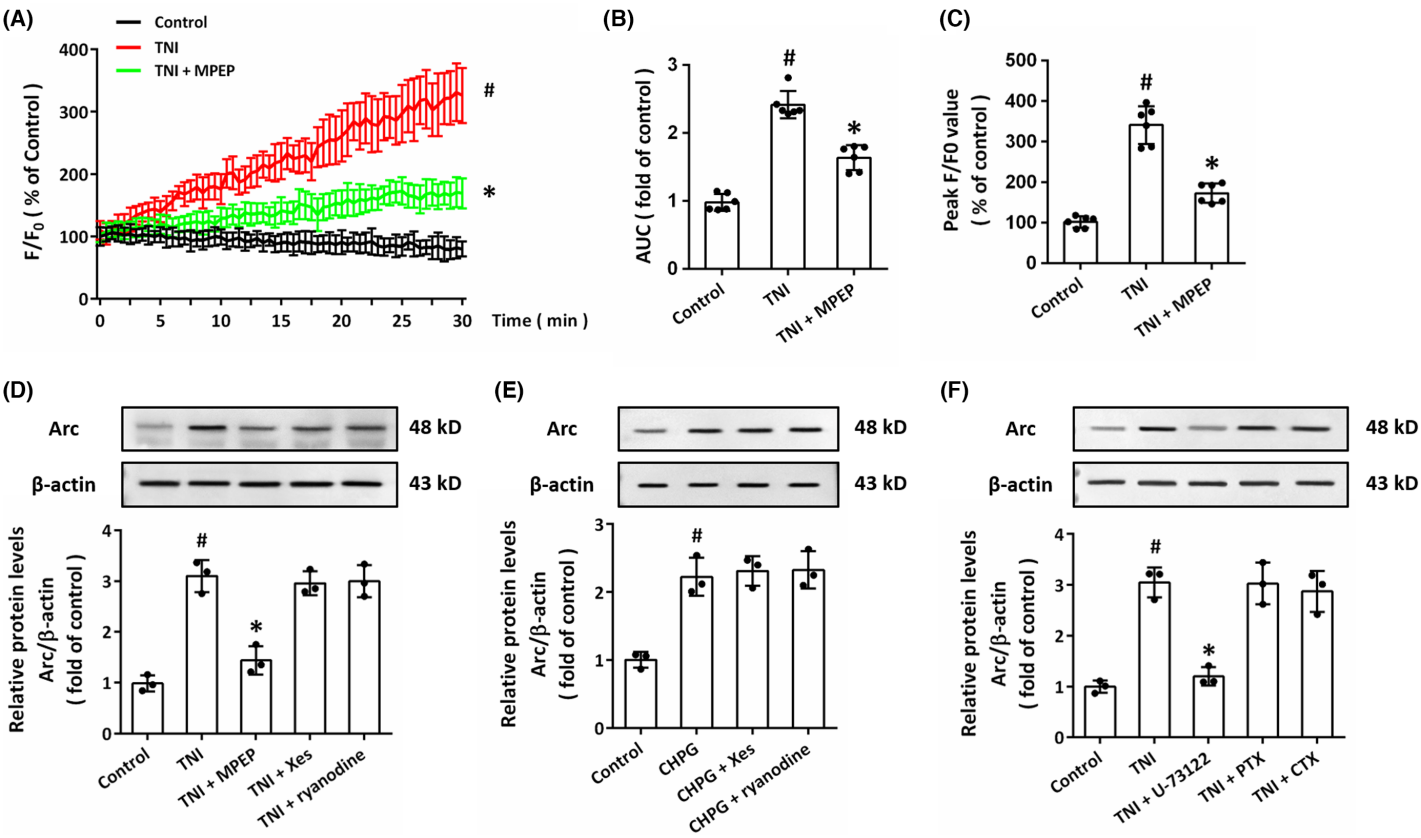
mGluR5 regulates Arc expression through G proteins. (A–C) Ca^2+^ imaging (A) and quantification (B, C) show that TNI induced an increase in intracellular Ca^2+^ via mGluR5 in cultured neurons. (D) Western blot and quantification show that the TNI‐induced upregulation of Arc was not associated with IP_3_R or RyR. (E) Western blot and quantification show that CHPG upregulated the levels of Arc, which was not associated with IP_3_R or RyR. (F) Western blot and quantification show that TNI upregulated Arc expression via G_q_ protein, but not via G_s_ or G_i_ protein. The data were represented as mean ± SEM. ^#^ *p* < 0.05 versus Control group and *p < 0.05 versus TNI group.

It is well‐known that mGluR5 participates in Ca^2+^ metabolism via regulating ER Ca^2+^ release. Thus, we measured intracellular Ca^2+^ concentration in neurons using Ca^2+^ imaging (Figure 3). The results showed that TNI induced significant increase in intracellular Ca^2+^, which was inhibited by the mGluR5 antagonist MPEP (Figure 3B). The peak value of *F*/*F*_0_ in MPEP treated group was much lower than that in TNI group (Figure 3C). The mGluR5‐associated ER Ca^2+^ release can be mediated by IP_3_R or RyR, but the antagonists of these receptors (Xes for IP_3_R or ryanodine for RyR) had no effect on the expression of Arc following TNI (Figure 3D). In addition, the induction of Arc protein induced by the mGluR5 agonist CHPG was not altered by Xes (2 μM) or ryanodine (25 μM) (Figure 3E). The mGluR5 belongs to the G protein‐coupled receptors family, and the G_i_ protein inhibitor pertussis toxin (PTX, 200 ng/mL), the G_s_ protein inhibitor Cholera toxin (CTX, 1 μg/mL) or the G_q_ protein inhibitor U‐73122 (3 μM) was used to determine the potential role of different types of G protein. As shown in Figure 3F, TNI‐induced Arc expression was significantly prevented by U‐73122, but not by PTX or CTX.

### 3.4 mGluR5 regulates Arc protein turnover

**FIGURE 4:**
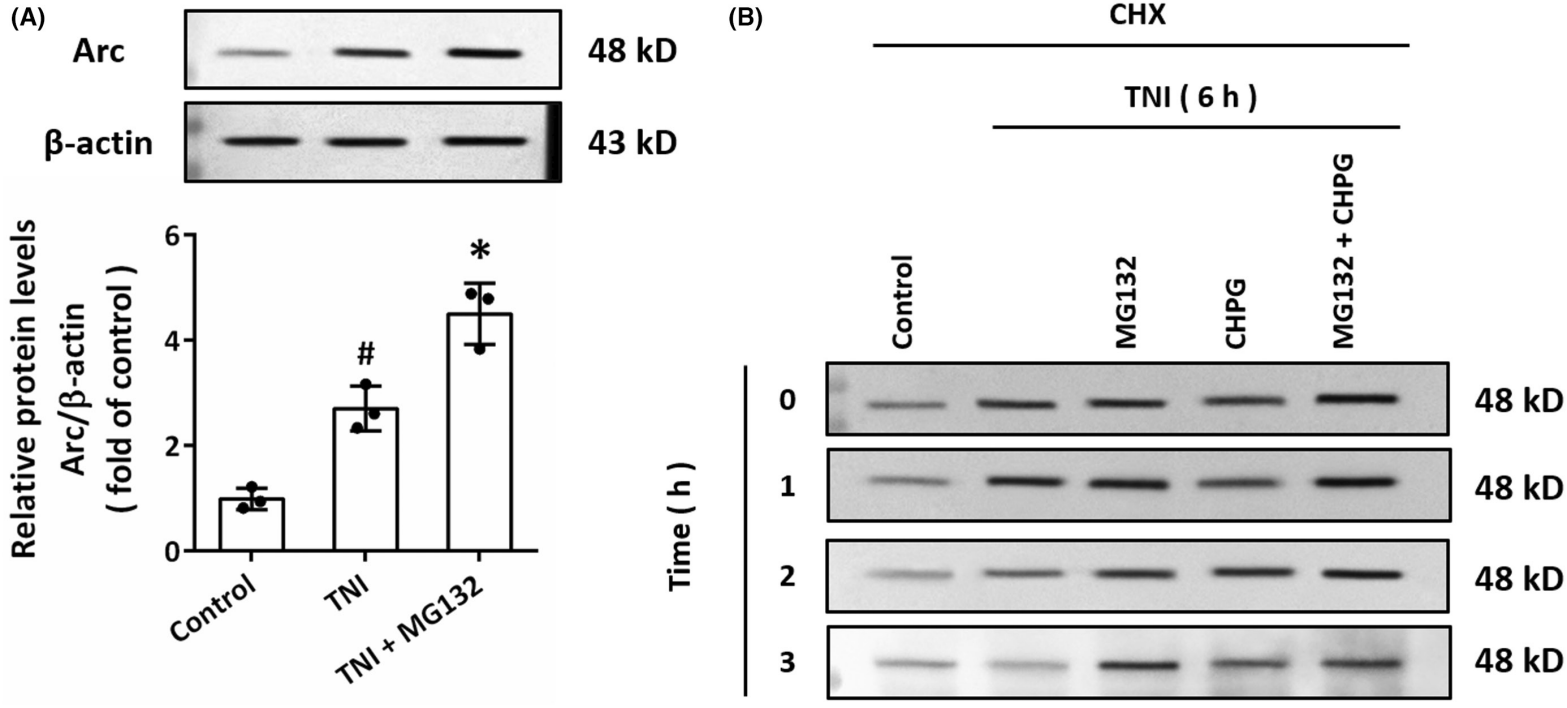
mGluR5 regulates Arc protein turnover. (A) Western blot and quantification show that TNI‐induced upregulation of Arc protein was potentiated by the proteasome inhibitor MG132. (B) Western blot shows that Arc protein within 6 h after TNI was stabilized by MG132 and CHPG after addition of CHX. The data were represented as mean ± SEM. ^#^ *p* < 0.05 versus Control group and *p < 0.05 versus TNI group.

The proteasome‐mediated Arc degradation has been reported as an important mechanism for regulating Arc expression. We found that the TNI‐induced increase in Arc protein levels were potentiated by the proteasome inhibitor MG‐132 (50 μM) (Figure 4A), indicating that Arc is a proteasome substrate. To investigate the role of mGluR5 in endogenous Arc protein degradation, we potentiated Arc expression by TNI, with MG‐132 and/or CHPG, and added CHX (10 μg/mL) to prevent further Arc synthesis. Then, the Arc protein levels were measured by western blot to 3 h thereafter (Figure 4B). The results showed that the Arc protein abundance declined more slowly in CHPG‐treated neurons than in control cells, suggesting that activation of mGluR5 inhibits the turnover of Arc protein.

### 3.5 Overexpression of Arc protects against TBI in vivo

**FIGURE 5:**
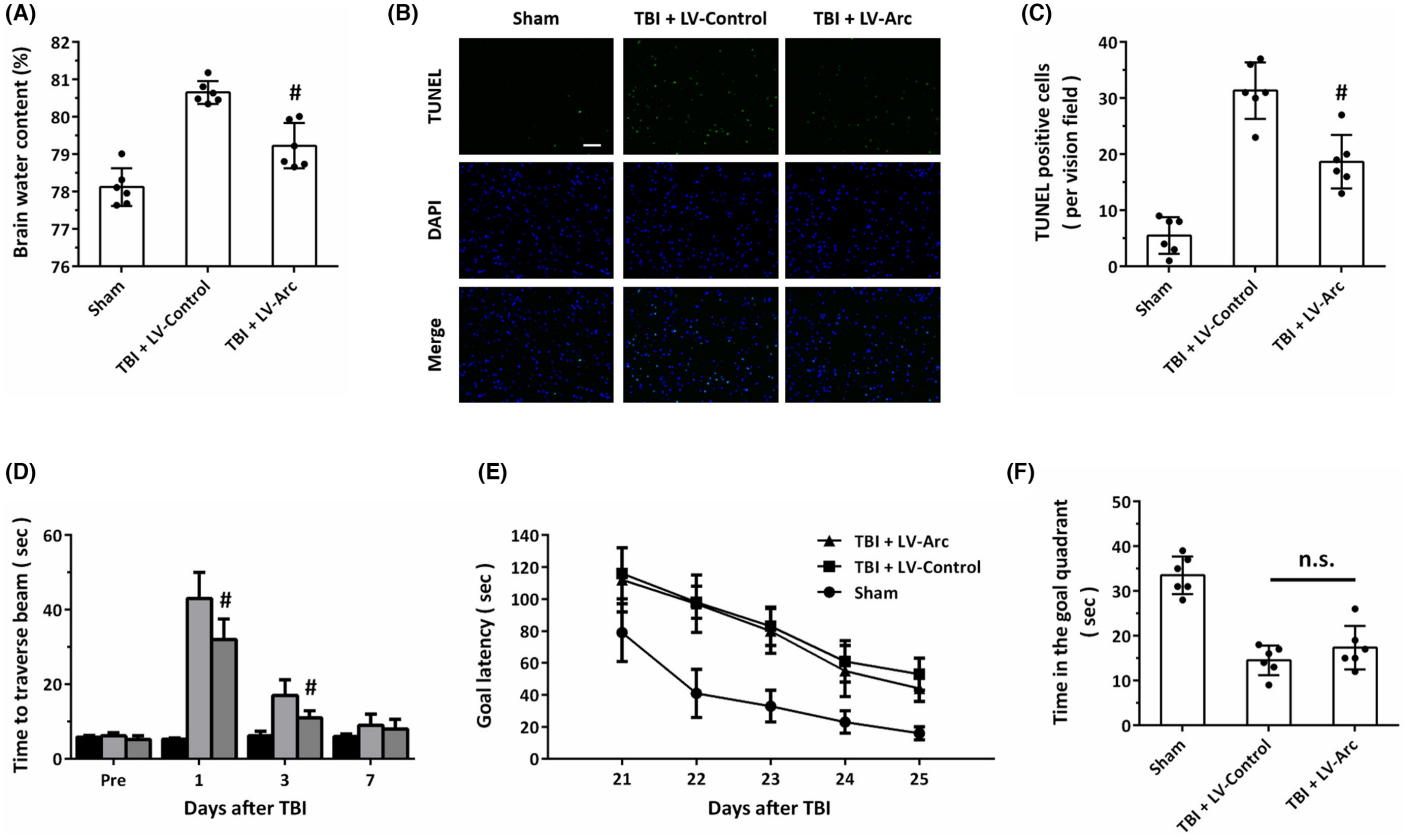
Overexpression of Arc protects against TBI in vivo. (A) Brain edema assay shows that overexpression of Arc attenuated brain water content at 24 h after TBI. (B, C) TUNEL staining (B) and quantification (C) show that overexpression of Arc inhibited apoptosis in the cortex at 24 h after TBI. Scale bar, 50 μm. (D) The beam walk task shows that the Arc overexpressed rats took significantly shorter time than LV‐Control group to traverse the beam. (E, F) MWM experiments show that no difference was found between LV‐Control and LV‐Arc groups in goal latency test at 21–25 post‐TBI (E) and probe trial following a 24‐h delay (F). The data were represented as mean ± SEM. ^#^ *p* < 0.05 versus LV‐Control group and n.s., not statistically different.

To investigate the role of Arc in vivo, lentivirus‐mediated overexpression of Arc was performed in a CCI model in rats. The results of brain water content assay showed that LV‐Arc significantly reduced brain edema after TBI (Figure 5A). We also detected apoptotic cells in brain sections using TUNEL staining (Figure 5B), and the results showed that the number of TUNEL‐positive cells in LV‐Arc group was lower than that in LV‐Control group (Figure 5C). Motor function after TBI was measured by the beam walk task, and the results showed that animals treated with LV‐Arc took significant shorter time than rats in LV‐Control group to traverse the beam at 1 and 3 days, but not 7 days, after TBI (Figure 5D). In addition, we also assayed cognitive deficits at 21–25 days after TBI using the MWM task. The results showed that TBI‐injured animals had significant longer goal latency, but there was no difference between TBI + LV‐Control group and TBI + LV‐Arc group (Figure 5E). As shown in Figure 5F, similar results were also observed in probe trial following a 24 h delay.

### 3.6 Overexpression of Arc protects against TNI in vitro

**FIGURE 6:**
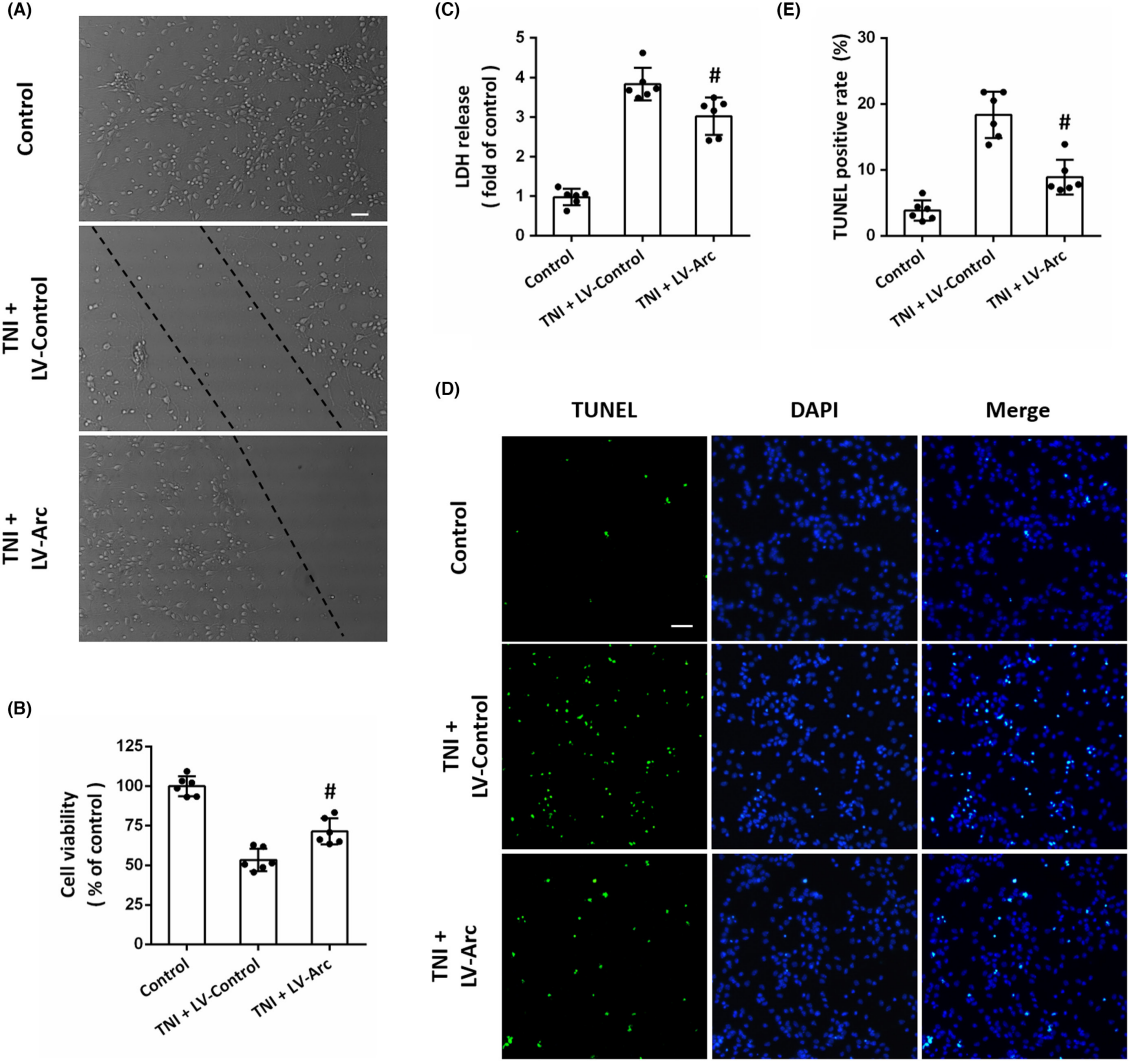
Overexpression of Arc protects against TNI in vitro. (A) Representative images of photomicrograph show the morphological changes of neurons following TNI. The dashed line indicates the edge of the scratch injury. Scale bar, 50 μm. (B) The cell viability assay shows that overexpression of Arc preserved neuronal viability at 24 h post‐TBI. (C) The LDH release assay shows that overexpression of Arc reduced neurotoxicity at 24 post‐TBI. (D, E) TUNEL staining (D) and quantification (E) show that overexpression of Arc attenuated apoptosis in neurons at 24 h post‐TBI. Scale bar, 50 μm. The data were represented as mean ± SEM. ^#^ *p* < 0.05 versus LV‐Control group.

To investigate the role of Arc in vitro, lentivirus‐mediated overexpression of Arc was performed in a TNI model in cortical neurons (Figure 6A). The results showed that the TNI‐induced decrease in cell viability was significantly reduced by transfection with LV‐Arc (Figure 6B). The cytotoxicity induced by TNI was assayed by measuring LDH release into the culture medium, and the results showed that the LDH level in TNI + LV‐Arc group was much lower than that in TNI + LV‐Control group (Figure 6C). In addition, we also detected apoptotic neurons using TUNEL staining (Figure 6D). As shown in Figure 6E, overexpression of Arc significantly attenuated the TNI‐induced neuronal apoptosis in vitro.

### 3.7 Arc mediates the protective effects of mGluR5 activation

**FIGURE 7:**
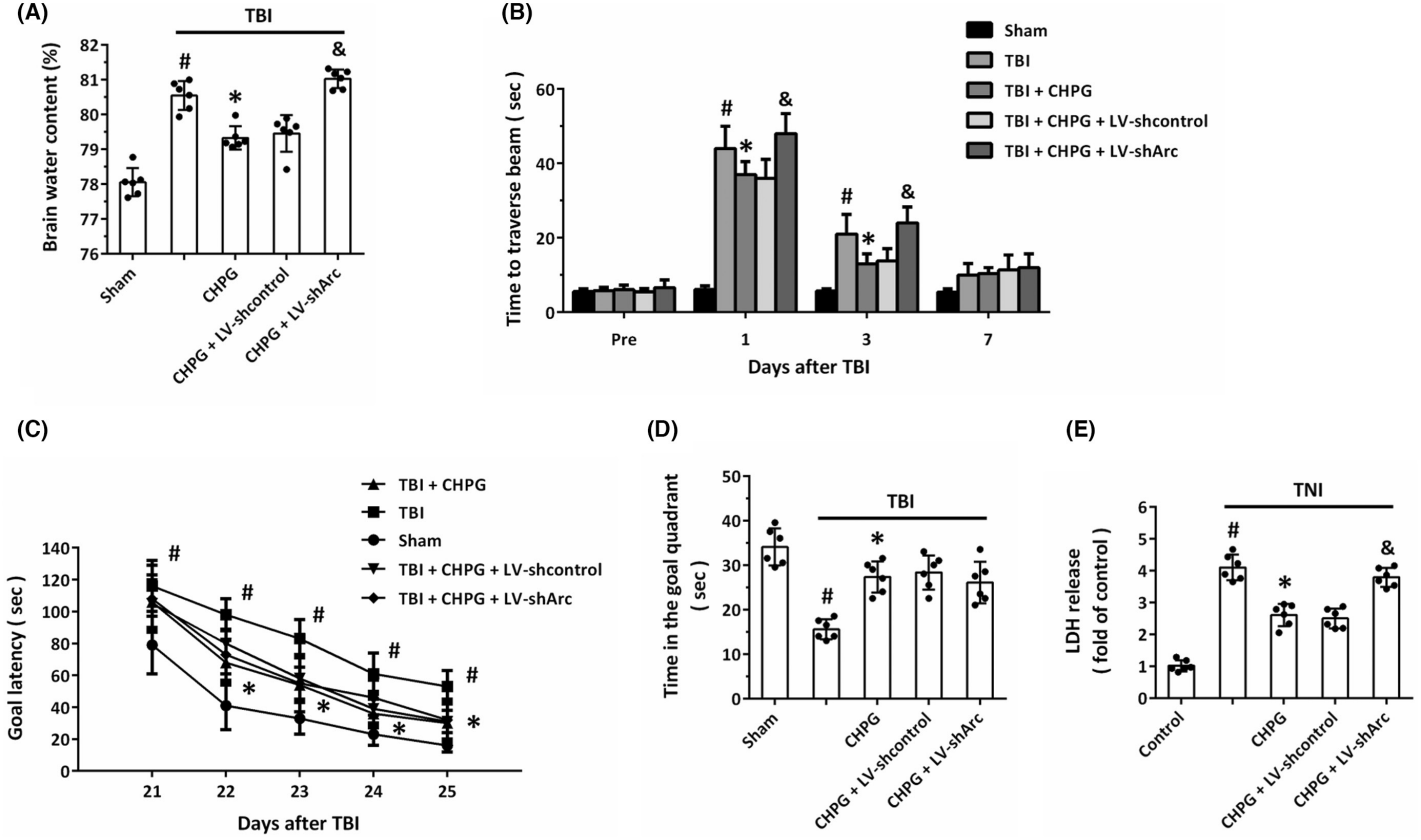
Arc mediates the protective effects of mGluR5 activation. (A) Brain edema assay shows that CHPG‐induced decrease in brain water content was prevented by Arc knockdown. (B) The beam walk task shows that CHPG‐treated rats took significantly shorter time than sham group to traverse the beam, but the rats transfected with LV‐shArc took significantly longer time than LV‐shcontrol group to traverse the beam. (C, D) MWM experiments show that CHPG‐treated rats had shorter goal latency (C) and spent a shorter time in the goal quadrant when compared with animals in sham group (D). No difference was found between CHPG+LV‐shcontrol and CHPG+LV‐shArc groups in goal latency test at 21–25 post‐TBI and probe trial following a 24‐h delay. (E) The LDH release assay shows that CHPG reduced LDH release at 24 h post‐TNI, which was reversed by Arc knockdown. The data were represented as mean ± SEM. ^#^ *p* < 0.05 versus sham or Control group, *p < 0.05 versus TBI or TNI group and ^&^ *p* < 0.05 versus CHPG+LV‐shcontrol group.

It has been reported that activation of mGluR5 exerts protective effects against TBI. Thus, we investigated the role of Arc in the protective effects of the mGluR5 agonist CHPG in our experimental TBI models. As expected, CHPG markedly reduced the TBI‐induced brain edema (Figure 7A) and motor dysfunction (Figure 7B), which were all partially reversed by Arc knockdown by LV‐shArc, as compared to LV‐shcontrol. In addition, CHPG significantly attenuated the cognitive deficits after TBI, evidenced by decreased goal latency (Figure 7C) and increased time in the goal quadrant (Figure 7D) from 22 to 25 days post‐TBI. Intriguingly, the CHPG‐induced protection on cognitive deficits was not altered by LV‐shArc or LV‐shcontrol transfection. As shown in Figure 7E, the CHPG‐induced attenuation of LDH release after TNI was also alleviated by Arc knockdown in vitro.

### 3.8 Arc mediates the mGluR5‐induced regulation of AMPAR signaling

**FIGURE 8:**
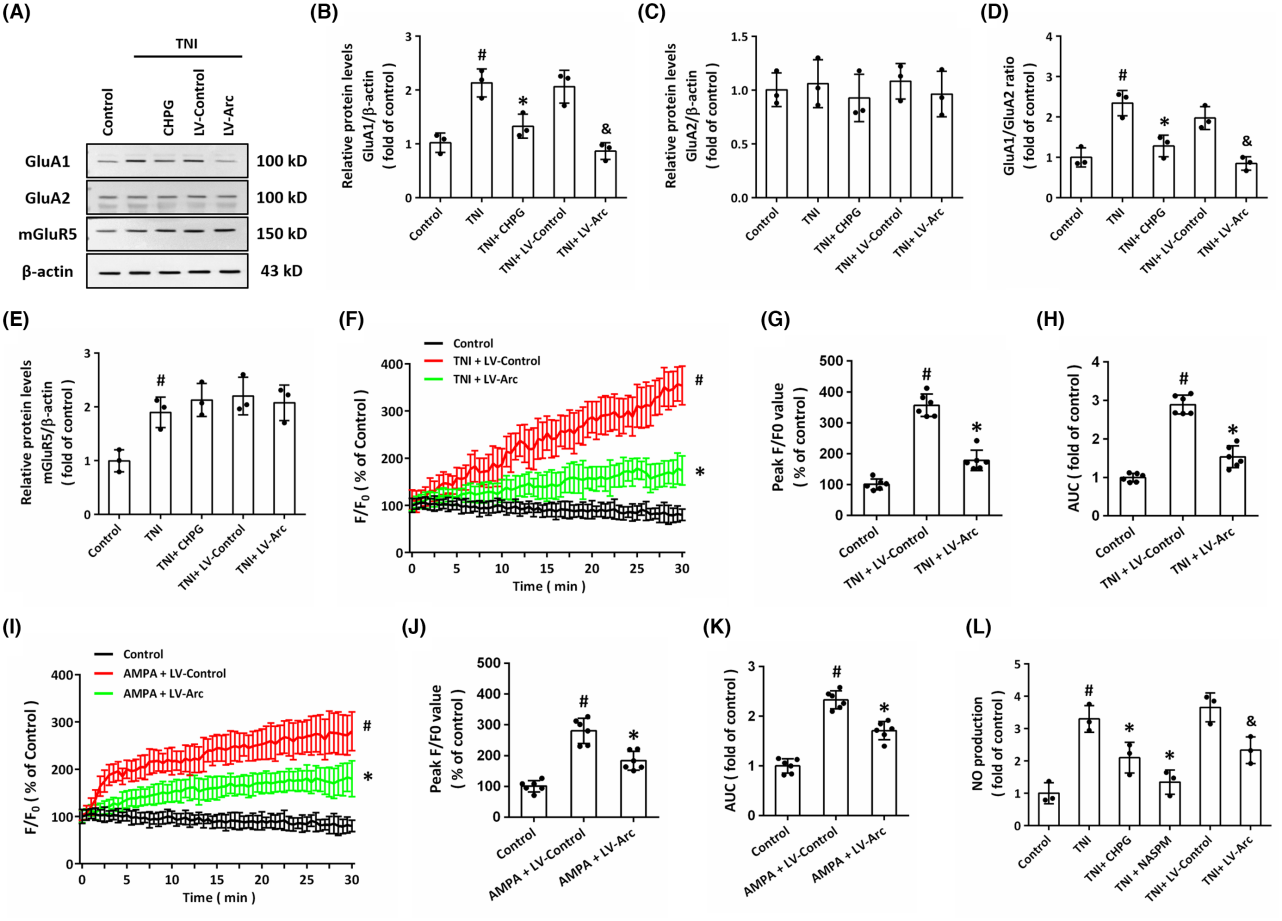
Arc mediates the mGluR5‐induced regulation of AMPAR signaling. (A–D) Western blot (A) and quantification (B–E) show that: TNI‐induced increase in GluA1 expression was partially prevented by CHPG or LV‐Arc (B); TNI, CHPG or LV‐Arc had no effect on GluA2 expression (C); TNI‐induced increase in GluA1/GluA2 ratio was partially prevented by CHPG or LV‐Arc (D); and TNI significantly increased the expression of mGluR5 in neurons (E). (F–H) Ca^2+^ imaging (F) and quantification (G, H) show that TNI‐induced increase in intracellular Ca^2+^ was partially prevented by Arc overexpression. (I–K) Ca^2+^ imaging (I) and quantification (J, K) show that AMPA‐induced increase in intracellular Ca^2+^ was partially prevented by Arc overexpression. (L) NO assay shows that TNI‐induced NO production was partially blocked by CHPG, NASPM or LV‐Arc transfection. The data were represented as mean ± SEM. ^#^ *p* < 0.05 versus Control group, *p < 0.05 versus TNI group and ^&^ *p* < 0.05 versus LV‐Control group.

Next, we performed western blot to detect the expression of mGluR5 and AMPAR subunits in our in vitro model (Figure 8A). TNI significantly increased the expression of GluA1, which was reduced by CHPG and Arc overexpression (Figure 8B). However, there was no difference in GluA2 expression between all examined groups (Figure 8C). Thus, the ratio of GluA1/GluA2 was changed as the same pattern as GluA1 in different groups (Figure 8D). As shown in Figure 8E, TNI increased the expression of mGluR5, while CHPG or LV‐Arc had no effect on mGluR5 levels. Next, we performed Ca^2+^ imaging to investigate the effect of Arc on intracellular Ca^2+^ metabolism (Figure 8F). The increased peak *F*/*F*_0_ value (Figure 8G) and AUC of Ca^2+^ concentration (Figure 8H) induced by TNI were both attenuated by LV‐Arc transfection as compared to LV‐Control group. In addition, we also investigated the effect of Arc on AMPA‐induced Ca^2+^ increase (Figure 8I). As shown in Figure 8J,K, overexpression of Arc significantly reduced intracellular Ca^2+^ increase after AMPA treatment. The excitotoxicity induced by AMPA has been shown to be associated with intracellular NO generation. Thus, we measured NO production in neurons, and the results showed that TNI markedly increased NO production, which was blocked by CHPG and the Ca^2+^‐permeable AMPAR antagonist NASPM (Figure 8L). The TNI‐induced NO generation was also attenuated by Arc overexpression.

### 3.9 Arc‐Homer1 interaction contributes to the mGluR5‐induced regulation of intracellular Ca^2+^ release

**FIGURE 9:**
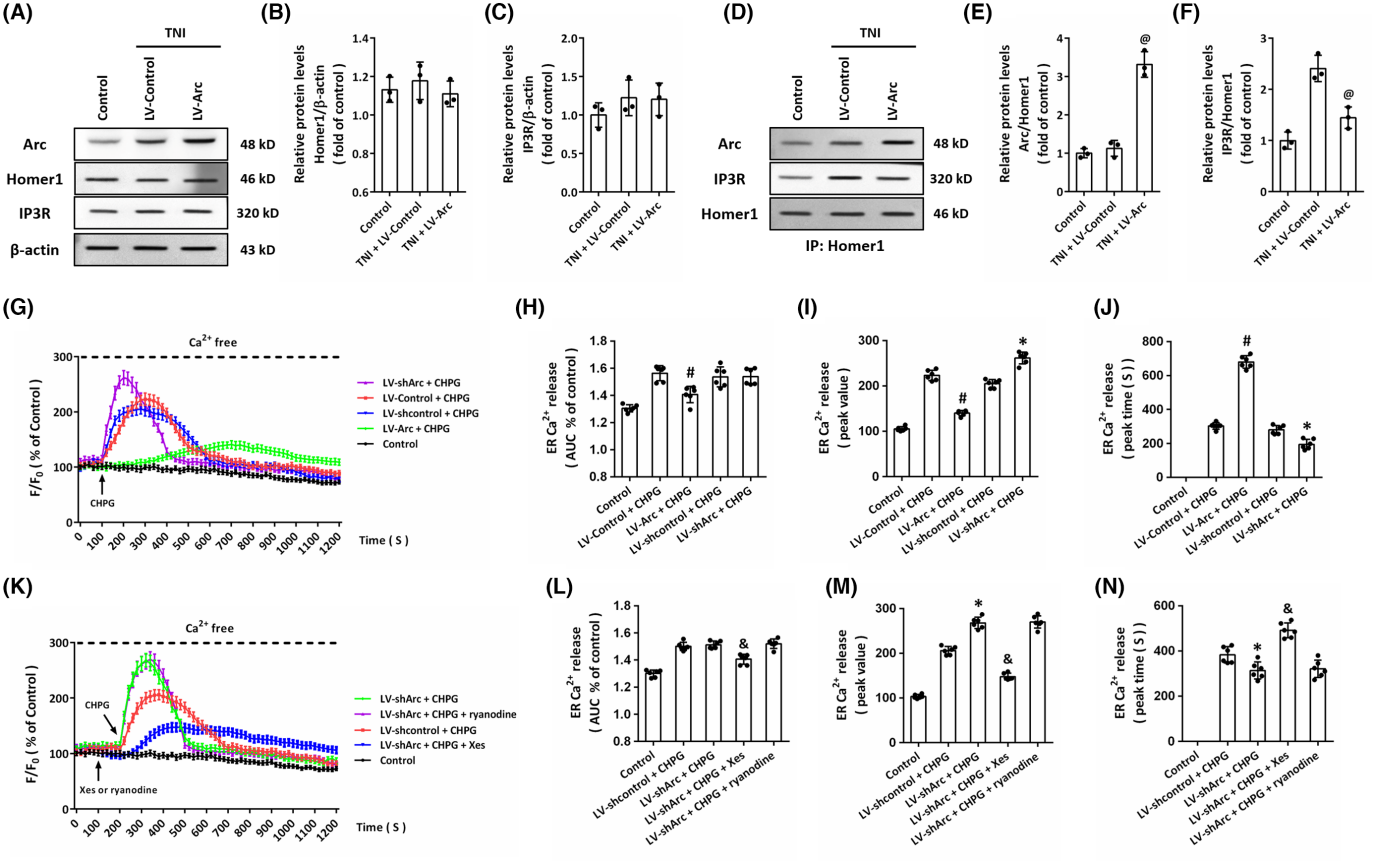
Arc‐Homer1 interaction contributes to the mGluR5‐induced regulation of intracellular Ca^2+^ release. (A–C) Western blot (A) and quantification (B, C) show that TNI or LV‐Arc had no effect on the expression of Homer1 (B) and IP3R (C). (D–F) Western blot after Homer1 immunoprecipitation show that overexpression of Arc significantly promoted the interaction of Arc and Homer1 (E) but disrupted the interaction of IP3R and Homer1 (F). (G–J) Ca^2+^ imaging in Ca^2+^ free solution with CHPG treatment (G) and quantification (H–J) show that: LV‐Arc reduced total ER Ca^2+^ release following CHPG treatment (H); LV‐Arc decreased peak value of ER Ca^2+^ release, and LV‐shArc increased peak value of ER Ca^2+^ release after CHPG treatment (I) and LV‐Arc extended the peak time of ER Ca^2+^ release, and LV‐shArc shortened the peak time of ER Ca^2+^ release following CHPG treatment (J). (K–N) Ca^2+^ imaging in Ca^2+^ free solution with CHPG treatment (K) and quantification (L–N) show that: CHPG‐induced ER Ca^2+^ release was attenuated by Xes, but not by ryanodine (L); LV‐shArc‐induced increase in peak value of ER Ca^2+^ release following CHPG treatment was revered by Xes, but not by ryanodine (M); and LV‐shArc‐induced decrease in the peak time of ER Ca^2+^ release following CHPG treatment was reversed by Xes, but not by ryanodine (N). The data were represented as mean ± SEM. ^@^ *p* < 0.05 versus TNI+LV‐Control group, ^#^ *p* < 0.05 versus LV‐Control+CHPG group, *p < 0.05 versus LV‐shcontrol+CHPG group and ^&^ *p* < 0.05 versus LV‐shArc+CHPG group.

The PSD family protein Homer1 mediates the mGluR5‐related intracellular Ca^2+^ release via IP_3_R. Thus, we performed western blot to detect the expression of Homer1 and IP_3_R (Figure 9A), and the results showed that neither TNI nor Arc overexpression had effect on Homer1 expression (Figure 9B). As sown in Figure 9C, similar results were also observed on IP_3_R expression. Homer1 is an important scaffold protein that facilitates interactions between PSD proteins by formation of post synaptic complex. Thus, we performed Co‐IP experiments using Homer1 antibody (Figure 9D). The interaction between Arc and Homer1 was observed in cortical neurons in vitro, which was significantly enhanced by Arc overexpression (Figure 9E). In contrast, the interaction between IP_3_R and Homer1 was markedly inhibited by LV‐Arc transfection when compared with LV‐Control group (Figure 9F).

Next, we repeated the Ca^2+^ imaging experiments in Ca^2+^ free medium to further investigate the role of Arc in intracellular Ca^2+^ release in cultured neurons (Figure 9G). CHPG caused an increase in intracellular Ca^2+^ concentration, and there was no difference between LV‐Control + CHPG and LV‐shcontrol + CHPG group in *F*/*F*_0_ trace. The CHPG‐induced Ca^2+^ release within 1200 s, as evidenced by AUC (Figure 9H), were markedly decreased by Arc overexpression, but not by LV‐shArc transfection. Arc overexpression reduced *F*/*F*_0_ peak value (Figure 9I) and delayed the *F*/*F*_0_ peak time (Figure 9J) in neurons after CHPG treatment. The opposite results were observed in LV‐shArc treated neurons as compared to LV‐shcontrol group after CHPG exposure. In addition, we used ryanodine and Xes to determine the potential involvement of related receptors (Figure 9K). The Ca^2+^ release induced by CHPG in Arc knockdown neurons was partially prevented by Xes but not by ryanodine (Figure 9L). As expected, the increase in *F*/*F*_0_ peak value (Figure 9M) and decrease in *F*/*F*_0_ peak time (Figure 9N) were both reversed by Xes, but not altered by ryanodine.

## 4 DISCUSSION

**FIGURE 10:**
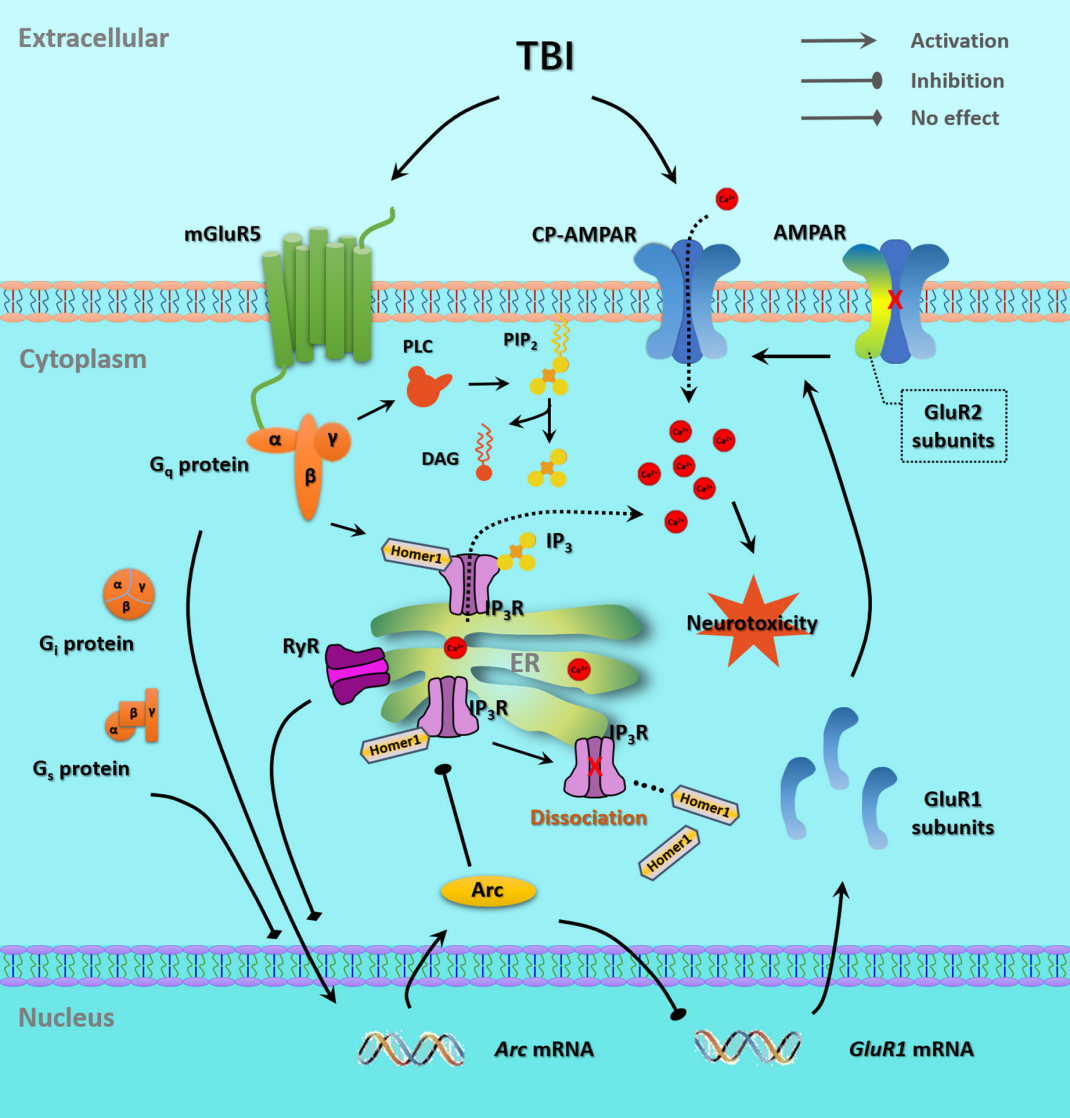
A proposed diagram tying together the various observations involved in the protection induced by mGluR5‐mediated Arc activation and Ca^2+^ signaling against experimental TBI. AMPAR, α‐amino‐3‐hydroxy‐5‐methylisoxazole‐4‐propionic acid receptor; CP‐AMPAR, Ca^2+^‐permeable AMPAR; DAG, diacylglycerol; ER, endoplasmic reticulum; IP_3_, inositol‐1,4,5‐trisphosphate; IP_3_R, inositol‐1,4,5‐trisphosphate receptor; mGluR5, metabotropic glutamate receptor 5; PIP_2_, phosphatidylinositol bisphosphate; PLC, phospholipase C; RyR, Ryanodine receptor; TBI, traumatic brain injury.

The molecular mechanisms of neuronal injury following TBI are complicated; and to date, there is no effective drug or treatment that can target the signaling pathway involved in the pathology in clinical practice. The present study identified the mGluR5‐mediated Arc activation as a protective mechanism that attenuated neurotoxicity following the experimental TBI through the regulation of intracellular Ca^2+^ hemostasis (Figure 10). We found that (a) experimental TBI increases Arc expression via mGluR5 in vivo and in vitro; (b) mGluR5 regulates Arc expression through G_q_ protein and protein turnover; (c) overexpression of Arc protects neuronal injury after TBI in vivo and in vitro; (d) Arc mediates the protective effects of mGluR5 activation following TBI; (e) Arc mediates the mGluR5‐induced regulation of AMPAR signaling and (f) Arc‐Homer1 interaction contributes to the mGluR5‐induced regulation of intracellular Ca^2+^ release. Due to the possible impact of exogenous gene overexpression techniques, the effect of endogenous Arc protein on neuronal injury in in vivo TBI model need to be further investigated.

As an IEG‐encoded protein, Arc is highly expressed in neurons, and is correlated with multiple forms of synaptic plasticity, including long‐term potentiation (LTP), long‐term depression (LTD) and homeostatic plasticity.^25^, ^26^ The expression of Arc mRNA and protein can be regulated by various receptor‐mediated mechanisms that drive up intracellular Ca^2+^ signaling and its downstream sequelae, especially by glutamate receptor cascades in postsynaptic excitatory synapses.^17^ However, the expression changes of Arc under pathological conditions are equivocal. It is a consensus that acute restraint stress increases Arc expression through glucocorticoid receptors (GRs) in frontal cortex, while both increases and no changes were reported in the hippocampus after acute stress simulation.^27^, ^28^, ^29^ In addition, for the effects of chronic stress on hippocampal Arc expression, one study showed an obvious increase, whereas another research reported a significant decrease.^30^, ^31^ Our previous study showed that glutamate at a toxic concentration markedly increased Arc mRNA and protein levels in primary cultured cortical neurons.^19^ In experimental subarachnoid hemorrhage (SAH) models, Arc protein levels were upregulated both in vitro and in vivo.^32^ More importantly, our recent results showed that TNI caused a temporal increase of Arc expression at 3 and 6 h.^20^ In this study, significantly increases in Arc protein levels were observed in both in vivo and vitro experimental TBI models, and increased Arc expression was mainly located at neurons. Thus, activation of Arc transcription and translation seem to be a common phenomenon under neurological disorders.

Many IEG‐coded proteins are found to be upregulated following neuronal injury, some of which are thought to activate downstream detrimental cascades, whereas the others are demonstrated to be endogenous protective mechanisms.^9^, ^33^, ^34^, ^35^ Our previous study showed that knockdown of endogenous Arc expression further promoted the TNI‐induced cytotoxicity and apoptosis,^20^ indicating the potential neuroprotective role of Arc. To test this hypothesis, lentivirus‐mediated gene overexpression was performed both in vitro and in vivo. Overexpression of Arc significantly reduced brain edema and neuronal apoptosis after TBI, which was in congruent with the in vitro data that Arc attenuated the TNI‐induced cytotoxicity and apoptosis. In addition, the beam balance task showed that the Arc‐overexpressed animals take shorter time to traverse beam at 1 and 3 days after TBI (but not at 7 days). However, no beneficial effect was observed as to MWM task, which showed that overexpression of Arc did not reduce the goal latency from 21 to 25 days after TBI. These data suggest that although Arc alleviates neuronal death and brain edema after TBI, it exerts no long‐term protection against neurological dysfunction. IEGs can be activated and transcribed by extrinsic signals within minutes after stimulation, and contributes to the quick response under pathological conditions, known as immediate‐early response (IER) processes.^36^ IEG protein products are commonly unstable and they are sometimes targeted for proteolytic degradation by the proteasome without prior ubiquitination.^37^ The expression and regulation pattern of IEGs indicate their potential role in acute phase of neurological dysfunction, and the IEG‐coded proteins, such as homer and preso, have been demonstrated to be involved in TBI‐related neurotoxicity.^16^, ^23^, ^38^

Expression, localization and stability of Arc are tightly associated with glutamate receptor signaling. Activation of NMDAR seems to be necessary to induce Arc transcription and localization,^39^ whereas AMPAR is thought to negatively regulate Arc transcription.^40^ Arc induction by brain‐derived neurotrophic factor (BDNF) depends on mitogen‐activated protein kinases (MAPK), which promotes Arc transcriptional activation via group I mGluRs.^41^, ^42^ Our previous study showed that glutamate caused rapid induction of Arc via NMDAR‐mediated phosphorylation of ERK and CREB.^19^ However, the role of group I mGluRs in TBI‐induced Arc expression has not been determined. By using mGluRs agonists and antagonists, our results showed that the TBI‐ and TNI‐induced Arc upregulation was nullified by inhibiting mGluR5 but promoted by the mGluR5 agonist CHPG, with no alteration following mGluA1 and group II mGluRs modulation. It has been demonstrated that knockdown of Arc prevented group I mGluRs from triggering AMPAR endocytosis or long‐term synaptic depression (LTD).^42^ Thus, the mGluRs signaling might be closely involved in both the upstream cascades that induce Arc expression and the complex physiological response that it produces. The role of group I mGluRs in TBI‐related brain damage and neurological dysfunction has been extensively studied, and previous studies and our data have shown that activation of mGluR5 via positive modulators exerted neuroprotective effects against TBI both in vitro and in vivo.^10^, ^11^, ^43^ Our present results showed that the CHPG‐induced protection against TBI and TNI were both partially prevented by Arc knockdown, indicating that Arc induction might be the downstream endogenous protective mechanism underlying the mGluR5‐induced protection against TBI.

Complicated crosstalk exists among distinct glutamate receptors, and activation of group I mGluRs results in a rapid redistribution of AMPAR.^44^ Considering our data that CHPG‐induced protection against TBI was dependent on Arc expression, and the fact that Arc plays a key role in determining synaptic strength through facilitation of AMPAR endocytosis,^45^ we speculated that regulation of AMPAR‐related toxicity might be the downstream mechanism of mGluR5‐induced neuroprotection. mGluR5 regulates the nitric oxide (NO)‐cGMP pathway in cerebellum via activation of AMPAR.^46^ Dennis et al. showed that oxygen and glucose deprivation (OGD) reduced the surface and total AMPAR protein levels by mGluR and adenosine A3 receptors.^47^ In the present study, the western blot assay showed that TNI increased the expression of GluA1 and the GluA1/GluA2 ratio, which were partially nullified by CHPG and Arc overexpression. AMPARs are highly dynamic receptors formed by the tetrameric assembly of four GluR receptors, GluA1‐4, and their functional properties largely depend on the composition of these subunits.^48^ It is well‐known that the GluA2 subunit critically controls Ca^2+^‐permeability, and the receptors lacking GluA2 are Ca^2+^ permeable, named as Ca^2+^‐permeable AMPAR.^49^ Thus, we further determined the intracellular Ca^2+^ metabolism using Ca^2+^ imaging, and the results showed that the TNI‐induced Ca^2+^ response and the AMPA‐induced Ca^2+^ increase in intracellular space were both significantly attenuated by transfection with LV‐Arc. Congruently, a previous study showed that synaptic mGluR activation drives plasticity of Ca^2+^‐permeable AMPAR, leading to dynamic changes in AMPAR‐mediated Ca^2+^ entry.^50^ Intriguingly, a recent study showed that a negative memory regulator MEF2 regulated the mGluR‐dependent AMPAR trafficking independently of Arc.^51^ In that paper, the authors found that MEF knockdown did not prevent the group I mGluRs agonist DHPG‐induced increase in Arc protein expression, but the relation between DHPG‐induced AMPAR trafficking and Arc expression was not determined.

Two major mechanisms are responsible for the intracellular Ca^2+^ overload in glutamate receptor‐related excitotoxicity following TBI: the iGluRs‐mediated Ca^2+^ influx from extracellular space and the intracellular Ca^2+^ release from ER via group I mGluRs.^52^, ^53^ The PSD family protein Homer1 mediates the mGluR5‐related intracellular Ca^2+^ release via IP_3_R, and our previous study showed that knockdown of the long‐form Homer1 protein, Homer1b/c, protected primary cultured cortical neurons against glutamate‐induced excitotoxicity via ER and mitochondrial pathway.^16^ In addition, downregulation of Homer1 was found to alleviate mitochondrial dysfunction through regulating Ca^2+^ homeostasis in cultured dopamine (DA) neurons.^54^ Therefore, we detected the expression of Homer1 and IP_3_R in our in vitro model after Arc overexpression, but so significant alterations of these two proteins was observed. Homer belongs to the most abundant scaffolding proteins in the PSD, and it works synergistically with shank to form a polymeric network structure for the interaction of downstream cascades.^15^ More recently, the Homer‐shank interactome was demonstrated to maintain the stable levels of excitability in neurons via regulating homeostatic scaling.^55^ Considering the results that overexpression of Arc attenuated the CHPG‐induced Ca^2+^ response in Ca^2+^ free condition, we speculated that Arc might regulate intracellular ER Ca^2+^ release via disturbing interaction between Homer1 and IP_3_R. In neuronal cells, Homer1 binds to the proline‐rich motifs PPXXF in the C‐terminus of mGluRs and the N‐terminus of the IP_3_R to mediate the agonist‐induced ER Ca^2+^ release.^56^ We performed Co‐IP assay to detect the Arc‐Homer1 and IP_3_R‐Homer1 interaction, and the results showed that overexpression of Arc nullified the enhanced IP_3_R‐Homer1 interaction induced by TNI. Moreover, the Arc downregulation‐induced aggravation of ER Ca^2+^ release following CHPG treatment in Ca^2+^ free medium was prevented by the IP_3_R inhibitor Xes, but not altered by ryanodine, the antagonist of another ER located Ca^2+^ channel RyR with the Homer‐binding motif.^57^, ^58^ All these data strongly suggest that the inhibition of IP_3_R‐Homer1 interaction and ER Ca^2+^ release partly contribute to the Arc‐induced regulation of Ca^2+^ metabolism.

## 5 CONCLUSION

In summary, the present study showed that the IEG‐coded PSD protein Arc was upregulated by TBI both in vivo and in vitro, which was dependent on the mGluR5 coupled G_q_ protein activation. This mGluR5‐mediated Arc induction might be an endogenous neuroprotective mechanism following TBI, and the AMPAR‐associated Ca^2+^ influx and ER Ca^2+^ release induced by Homer1‐IP_3_R pathway were involved in this protection.

## CONFLICT OF INTEREST STATEMENT

The authors declare that they have no conflict of interest.

## Supporting information


**Data S1.**.

## Data Availability

The data used to support the findings of this study are available from the corresponding author upon request.
