# Supplementary material for: The mGluR5‐mediated Arc activation protects against experimental traumatic brain injury in rats
Source: CNS Neurosci Ther. 2024 Aug 6;30(8):e14695. doi: 10.1111/cns.14695 (PMC11303269; doi:10.1111/cns.14695)

Figure 1A-Arc

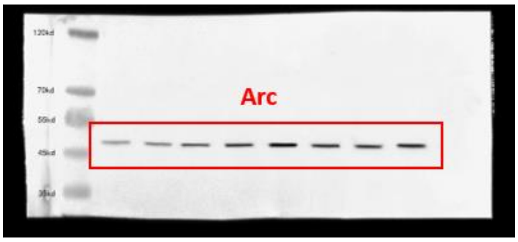

Figure 1A-β-actin

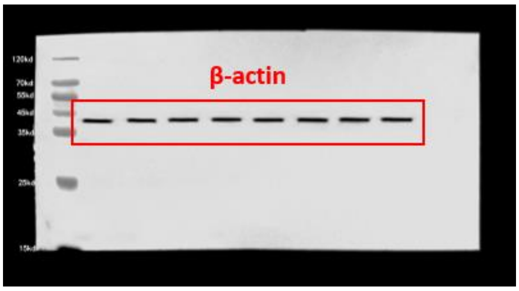

Figure 1C-Arc

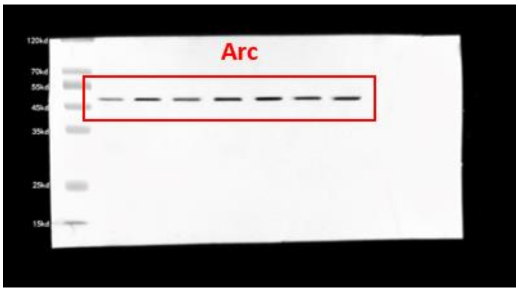

Figure 1C-β-actin

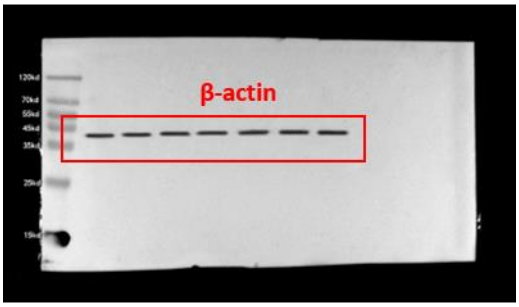

Figure 2A-Arc

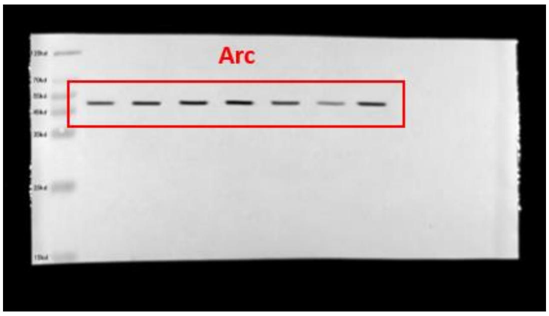

Figure 2A-β-actin

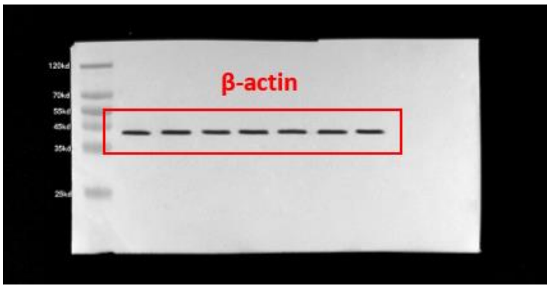

Figure 2C-Arc

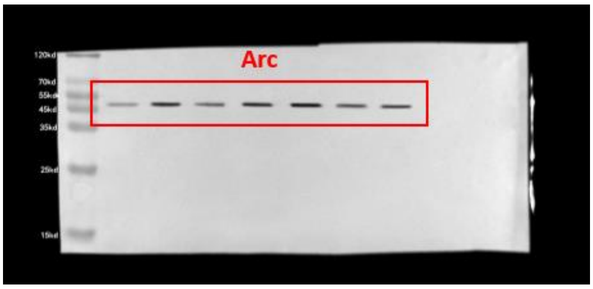

Figure 2C-β-actin

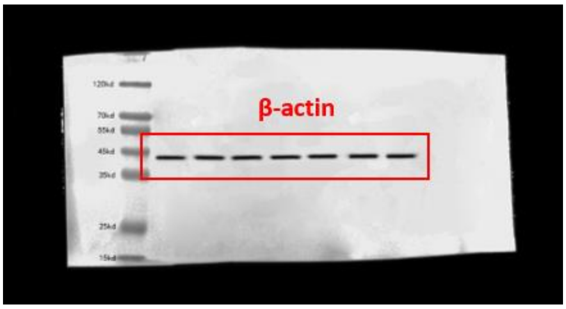

Figure 3D-Arc

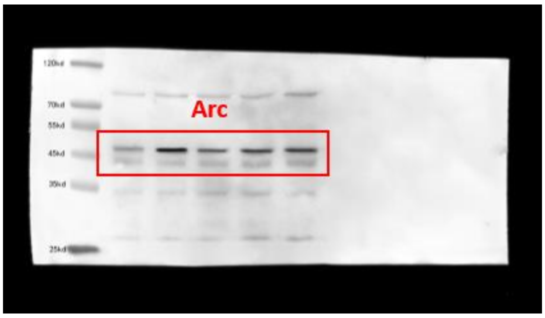

Figure 3D-β-actin

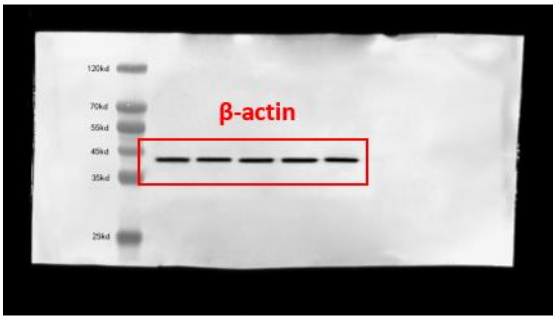

Figure 3E-Arc

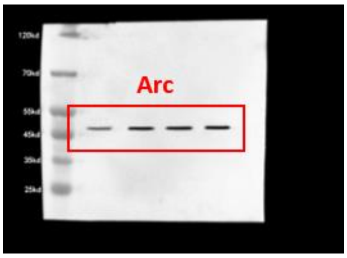

Figure 3E-β-actin

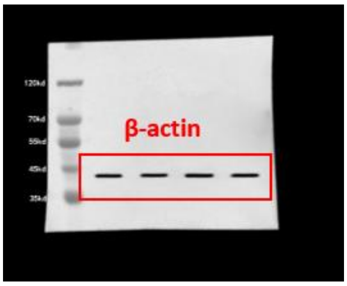

Figure 3F-Arc

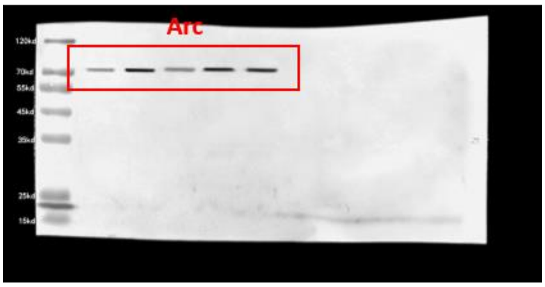

Figure 3F-β-actin

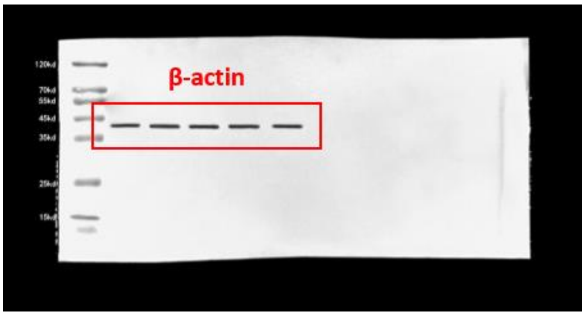

Figure 4A-Arc

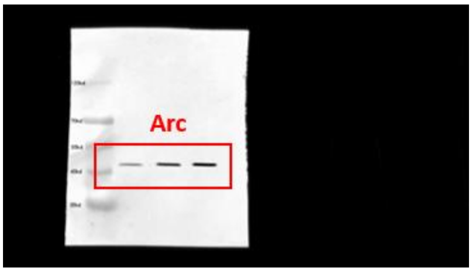

Figure 4A-β-actin

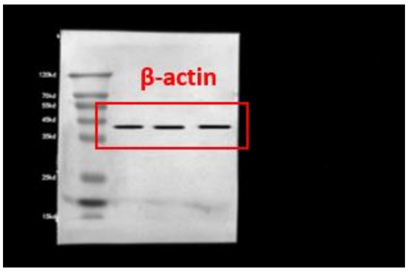

Figure 4B-Arc-0h

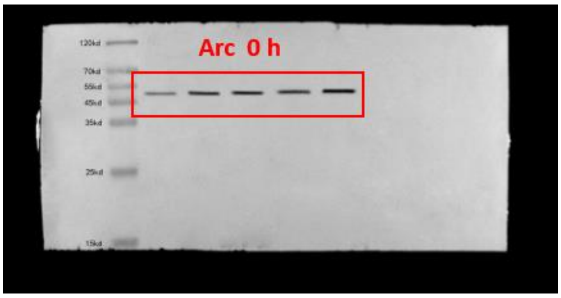

Figure 4B-Arc-1h

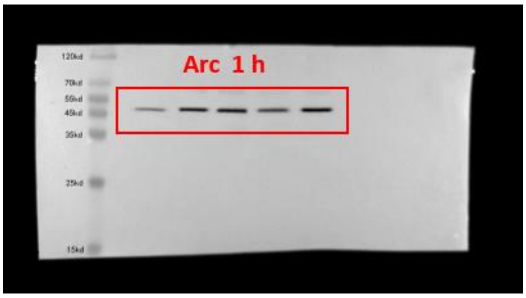

Figure 4B-Arc-2h

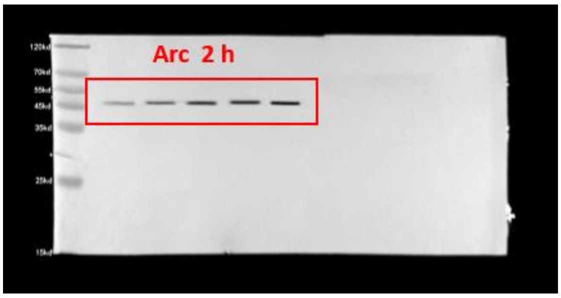

Figure 4B-Arc-3h

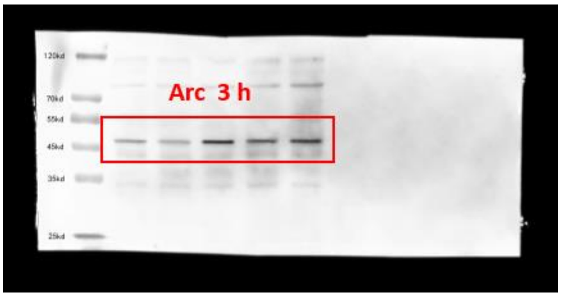

Figure 8A-GluA1

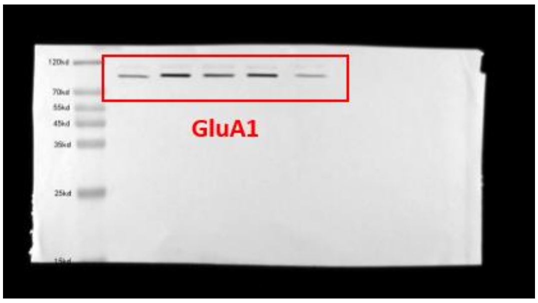

Figure 8A-GluA2

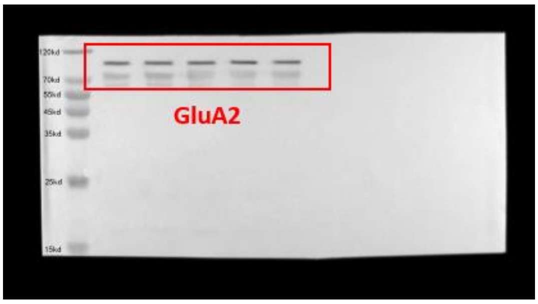

Figure 8A-mGluR5

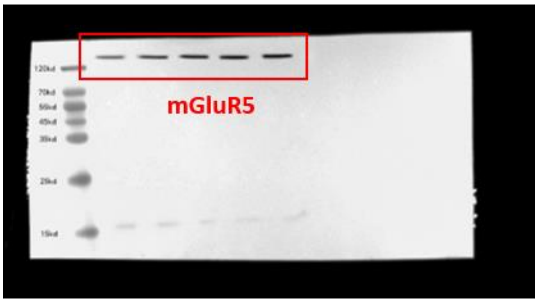

Figure 8A-β-actin

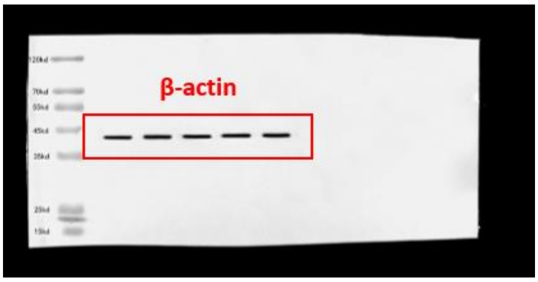

Figure 9A-Arc

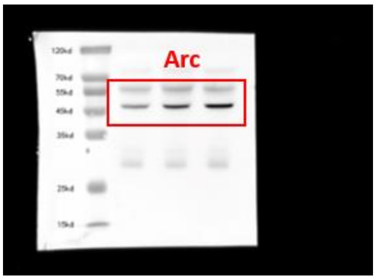

Figure 9A-Homer1

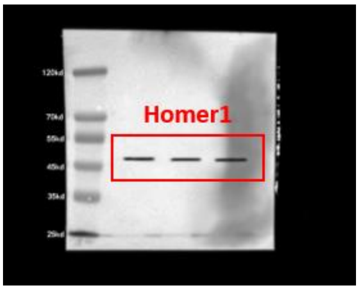

Figure 9A-IP3R

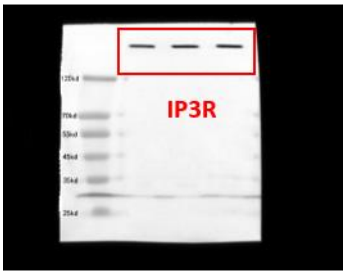

Figure 9A-β-actin

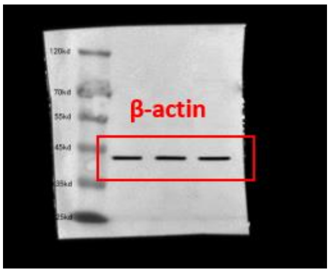

Figure 9D-Arc

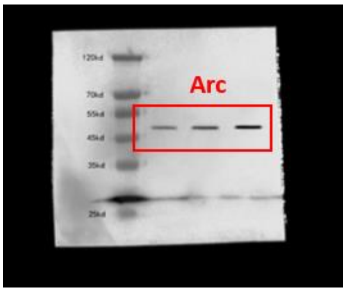

Figure 9D-IP3R

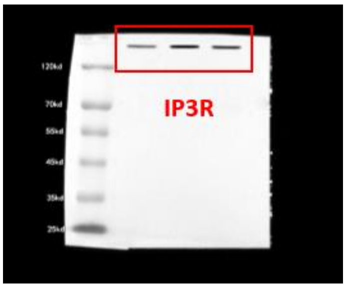

Figure 9D-Homer1

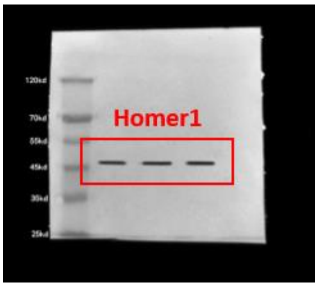

Supplement: Supplementary file 1 — Data S1.. [file CNS-30-e14695-s001.pdf]
